# Supplementary material for: Changing parental feeding practices through web-based interventions: A systematic review and meta-analysis
Source: PLoS One. 2021 Apr 28;16(4):e0250231. doi: 10.1371/journal.pone.0250231 (PMC8081248; doi:10.1371/journal.pone.0250231)
Supplement: S1 Table — Legend: CG = control group and IG = intervention group. (DOCX) [file pone.0250231.s003.docx]

**S1 Table. Summary of parental web-based interventions that assessed parental feeding practices as outcome.**

| **Author, year, country, name of the program** | **Study objectives** | **Participants** | **Variables measured** | **Intervention and control description** |
| --- | --- | --- | --- | --- |
| Byrd-Bredbenner et al. (2017)  USA  *HomeStyles* | To examine the efficacy of the intervention through an RCT (vs. an attention control condition), on the weight-related aspects of the home environment and lifestyle behavioral practices of families. | Parents of preschool children (2 to <6 years)  **IG**: N=252 (analyzed: N=89)  **CG**: N=237 (analyzed: N=83) | **Measurement time points**: baseline, immediately after the intervention  **Parental feeding practices**: food-related lifestyle practices (family meal frequency, family meal location, media device use at family meals, TV use at family meals and snacking occasions, family mealtime emotional environment, family meal planning, parent modeling of healthy eating behaviors); household food availability (fruits/vegetables, salty/fatty snacks, sugar-sweetened beverages, breakfast foods, milk)  **Other**: parent and child health status; parent and child height and weight; parent and child dietary, screen time, sleep, and physical activity behaviors; parent self-efficacy for meal preparation and promoting healthy eating behaviors; parent self-efficacy for food-related childhood obesity-protective practices; physical activity-related lifestyle practices; parent self-efficacy for physical activity-related childhood obesity-protective practices; parent values related to obesity protective practices; physical activity environment; home media environment | **Intervention objectives:** Improve home environment and lifestyle behavior practices (diet, sleep, physical activity) to promote optimal child’s growth and prevent obesity.  **IG** (*Healthy HomeStyles*): Twelve, 4-page mini-magazines focusing on key nutrition, physical activity or sleep messages. Parents were instructed to spend about 15 minutes reviewing each guide, setting goals and taking steps to implement the goals in the next few weeks. Parents also received specific nudges 4 to 5 days after accessing the guide. The use of each guide was estimated to take 16 to 30 days.  **CG** (*Safe* *HomeStyles*): Twelve mini-magazines with content about indoor air quality, household poisons, home and food safety. |
| Cullen, Thompson & Chen (2017)  USA  *Family Eats* | To evaluate the efficacy of the intervention through a randomized clinical trial (vs. a minimal intervention control condition), regarding the promotion of healthy home food environments and positive parental behaviors for African American families. | Parents of older children (8 to 12 years), African American families  **IG**: N=92 (analyzed: N=61)  **CG**: N=34 (analyzed: N=25) | **Measurement time points**: baseline, immediately after and 4 months after the intervention  **Parental feeding practices**: home availability (reported by parents and children: 100% fruit juice, fruit, vegetables, sugar-sweetened beverages; whole milk, low-fat milk, low fat/fat-free foods); family food preparation practices (meat modification, reduced fat, substitution, fruit vegetables, menu planning skills, healthy eating out); parental modeling (reported only by children)  **Other**: parent and children height and weight; dietary behaviors (reported by parents and children: 100% fruit juice, fruit, vegetables, fruits + vegetables, soda or pop, milk); parent self-efficacy regarding fruit and vegetables planning and serving, modeling consumption and availability | **Intervention objectives:** Promote healthy home food environment and positive parental behaviors, to improve dietary behaviors of the family.  **IG** (*Family Eats*): Eight stories about the Johnson family during their attempt to change family eating habits. In each session, parents viewed a story, were asked to complete a challenge (goal) during the following week, and to give their opinion (through a poll) about how to solve a specific family problem. Several materials were available on the website (recipes, tip sheets targeting the sessions).  **CG**: Access to the website in the same eight weekly sessions. Graphic stories were replaced with a single short paragraph about the target of the session. The challenge (goal) information and the family food problems were also not available. |
| Duncanson, Burrows & Collins (2016)  Australia  *Feeding Healthy Food to Kids* | To evaluate the efficacy of the intervention through a RCT (vs. minimal intervention control condition) on child feeding and parenting style.  To measure the stability of child feeding practices and parenting style dimensions over 12 months.  To test a composite child feeding score and a composite parenting style score. | Parents of preschool children (2 to <6 years), rural setting families  **IG**: N=75 (analyzed: N=75)  **CG**: N=71 (analyzed: N=71) | **Measurement time points**: baseline, 3 months and 12 months after the intervention  **Parental feeding practices**: child feeding practices (restriction, pressure to eat, monitoring)  **Other**: perceived responsibility; parenting style (warmth, inductive reasoning, parent efficacy, self-efficacy, overprotection, hostility) | **Intervention objectives:** provide parents with nutrition resources, to improve children’s eating patterns  **IG (***Feeding Healthy Food to Kids)*: *Tummy Rumbles* interactive CD and *Raising Children* DVD specific modules and resources, accessed over 9 months, for at least one hour each during the intervention period. The Tummy Rumbles is an interactive nutrition education CD divided into 10 modules about the five food groups, dietary fats, fussy eaters, healthy lunchbox ideas, food budgeting and reading food labels, while Raising Children is a guide to parenting from birth to 5 years.  **CG**: Delivery of a generic nutrition brochure and the *Active Alphabet* physical activity resource. |
| Frenn et al. (2013)  USA  *Project FUN* | To evaluate the feasibility and efficacy of parent-and/or child-focused online interventions through a cluster randomized control trial (vs. an evaluation only control condition), examining the relationships of changes in parents’ and child’s variables to changes in child’s nutrition-related outcomes (BMI, physical activity and dietary fat intake). | Parents of older children (5^th^, 7^th^ and 8^th^ grade students)  **Four arms**:  - parent and child intervention condition: N=15 (analyzed: N=10)  - parent instruments and child online condition: N=18 (analyzed: N=17)  - parent online and child instruments condition: N=13 (analyzed: N=8)  - parent and child instruments N=16 (analyzed: N=15) | **Measurement time points**: baseline, 6-9 months after  **Parental feeding practices**: food parenting practices (reported by parents and children: monitoring of sweets and high-fat foods, restriction of food, pressure to eat); family support for reduction of dietary fat (reported only by children)  **Other**: parent BMI and child BMIp; dietary fat and physical activity (reported only by children); family support for sedentary activity and increased physical activity (reported only by children); food and activity parenting practices (reported by parents and children: concern about child/their own overweight; responsibility for portions, type and kinds of foods; perceived control of foods; exercise monitoring, support and control); parent modeling of activity; parental food scan. | **Intervention objectives:** develop authoritative parenting approaches, family support and parental modeling, to improve children’s diets  **Intervention conditions:**  **-** *Parent and child online*: Children completed eight modules promoting healthy nutrition and physical activity over a period of 3-4 weeks. Intervention included four 2-3 minute videos with child actors illustrating the concepts and it took 10-30 minutes to finish each of the modules. Six parental modules were designed to develop authoritative parenting approaches, family support and parental modeling. Each module could be completed in 5-10 minutes. Links to family physical activity opportunities and healthy recipes in both the child and adult interventions were provided.  - *Parent instruments and child online*: Children’s intervention only  - *Parent online and child instruments*: Parental intervention only  - *Parent and child instruments*: Completion of the evaluation protocol. |
| Knowlden et al. (2015)  USA  *EMPOWER* | To evaluate the efficacy of the intervention through an RCT (vs. an active control condition) regarding four child behaviors (physical activity, fruit and vegetable intake, sugar-free beverages intake, and screen time) and five maternal-facilitated constructs of Social Cognitive Theory (environment, emotional coping, expectations, self-control and self-efficacy). | Mothers of preschool children (4 to 6 years)  **IG**: N=29 (analyzed: N=25)  **CG**: N=28 (analyzed: N=25) | **Measurement time points**: baseline, midpoint (between the 4^th^ and 5^th^ sessions), immediately after the intervention  **Parental feeding practices**: maternal-facilitated environment for child fruit and vegetable consumption; maternal-facilitated environment for sugar-sweetened beverage intake  **Other**: maternal-facilitated emotional coping, expectations, self-control, self-efficacy for: physical activity, fruit and vegetable consumption, sugar-free beverage consumption and screen time; maternal-facilitated environment for: physical activity, and screen time | **Intervention objectives:** improve 5 constructs in mothers, environment, emotional coping, expectations, self-control, and self-efficacy, to improve child physical activity, fruit and vegetable intake, sugar-free beverage intake, screen time  **IG** (*EMPOWER*): Five educational sessions, each covering one of the four child behaviors, occurring on a weekly basis, and one booster session delivered in week 6 (about nutrition and physical activity). Each session included 10 to 15-minute audiovisual presentation, an interactive worksheet and a discussion board post.  **CG** (*Healthy Lifestyles*): Five sessions with a knowledge-based intervention, which delivered general health knowledge regarding the same four behaviors, but did not focus on modification of SCT constructs. |
| Knowlden & Sharma (2016)  USA  *EMPOWER* | To determine the effects of the intervention at 1-year, post-intervention follow-up, regarding the four child behaviors and the five maternal-facilitated constructs defined earlier. | Mothers of preschool children (4 to 6 years)  **IG**: N=29 (analyzed: N=22)  **CG**: N=28 (analyzed: N=22) | **Measurement time points**: baseline, midpoint (between the 4^th^ and 5^th^ sessions), immediately after, and 12 months after the intervention  **Parental feeding practices**: the same  **Other**: the same | The same |
| Knowlden & Conrad (2018)  USA  *EMPOWER* | To determine the effects of the intervention at the 2-year post-intervention follow-up, regarding the four child behaviors and the five maternal-facilitated constructs defined earlier. | Mothers of preschool children (4 to 6 years)  **IG**: N=29 (analyzed: N=19)  **CG**: N=28 (analyzed: N=18) | **Measurement time points**: baseline, midpoint (between the 4^th^ and 5^th^ sessions), immediately after, 12 months and 24 months after the intervention  **Parental feeding practices**: the same  **Other**: the same | The same |
| Hammersley et al. (2019)  Australia  *Time2bHealthy* | To examine the efficacy of the intervention through a RCT (vs. an active control condition) on child’s BMI, obesity-related behaviors, parent modeling, and parent self-efficacy. | Parents of preschool children (2 to 5 years) who are overweight, or at or above 50^th^ BMI percentile for age and sex.  **IG**: N=42 (analyzed: N=42)  **CG**: N=44 (analyzed: N=42) | **Measurement time points**: baseline, immediately after, and 3 months after the intervention  **Parental feeding practices**: child-feeding practices (restriction and pressure to eat), parent modeling  **Other**: child BMI, child dietary intake (daily fruit and vegetables intake, fruit juice and sugary drinks, discretionary foods intake, kJ per kg of body weight, percentage of kJ from sugar and saturated fat); physical activity, screen time and sleep behaviors; parent self-efficacy regarding nutrition, physical activity, screen time and sleep | **Intervention objectives:** improve parental self-efficacy and modeling regarding child’s nutrition, physical activity, sleep, screen time, to facilitate child’s behavior change  **IG** (*Time2bHealthy*): 11-week program with six modules about four child’s obesity-related behaviors: nutrition, physical activity, screen time and sleep. Each module comprised reading material, videos, activities and quizzes. A goal-setting component was included, and parents received feedback and other advices from a dietitian. Parents were encouraged to participate in a closed Facebook group and exchange personal experiences and ideas about the process of change. At the end of the program, parents also received fortnightly emails with infographics summarizing the key points of each module, until the 6-month follow-up.  **CG**: Fortnightly emails with links to Raising Children Network website. The topics were similar to IG modules, and included general health information without the interactive component. |
| Helle et al. (2019a)  Norway  *Early Food For Future Health* | To evaluate the efficacy of the intervention through a RCT (vs. a treatment as usual control condition) on parental feeding practices and infant eating behaviors at 12 months old children. | Parents of infants (3 to 5 months)  **IG**: N=360 (analyzed: N=236-269)  **CG**: N=358 (analyzed: N=219-264) | **Measurement time points**: baseline and immediately after the intervention  **Parental feeding practices**: mealtime routines (child eating the same for dinner; parents making separate dinner for the child; child is sitting at the dinner table when eating; child playing or watching TV/tablet while eating); child eating meal together with family; maternal feeding practices (awareness of infant satiety and hunger clues, using food to calm fussiness, feeding on schedule)  **Other**: child eating behaviors; child food intake (fruit and vegetables, non-core foods/drinks, commercially prepared dinner, homemade dinner) and variety of fruit and vegetables tasted; child anthropometry; concern about infant under-eating and being underweight; concern about infant over-eating and being overweight; decisions on what and how much child should eat; neophobia | **Intervention objectives:** promote parental feeding practices to promote infant’s healthy eating behaviors  **IG** (*Early Food For Future Health*): Seven monthly video clips of 3 to 5 minutes duration, focusing on infant feeding topics (e.g., development of taste preferences) together with the corresponding recipes and cooking-films  **CG**: Routine care from their local child health clinic with regular consultations at child age 6, 8, 10 and 12 months. Consultations include measurements of weight and length, and conversation related to the child’s health, growth and psychomotor development. |
| Helle et al. (2019b)  Norway  *Early Food For Future Health*  First trial study: Helle et al., 2019a. | To evaluate the effects of the intervention at child age 24 months (one year after the intervention) on parental feeding practices and style, and infant eating behaviors. | Parents of infants (3 to 5 months)  **IG**: N=360 (analyzed: N=152-178)  **CG**: N=358 (analyzed: N=143-165) | **Measurement time points**: baseline and immediately after, and 12 months after the intervention  **Parental feeding practices**: mealtime routines (child eating the same for dinner; child is sitting at the dinner table when eating; child playing or watching TV/tablet while eating); child eating meal together with family; maternal feeding practices (restriction and pressure)  **Other**: child eating behaviors; child food intake (vegetables, fruits, sweet/salty snacks, soft-drinks); child anthropometry; maternal feeding styles; neophobia | The same |
| Ledoux et al. (2018)  EUA  *Happier Meals* | To evaluate the efficacy of the intervention through a RCT (vs. a minimal intervention control condition) regarding the promotion of responsive feeding practices and the division of responsibility between parents and children in feeding context.  To evaluate parents’ ability to understand the contents of the video and to identify with the characters.  To determine the parents’ acceptability of the video. | Parents of preschool children (2 to 5 years)  **IG**: N=32 (analyzed: N=32)  **CG**: N=18 (analyzed: N=18) | **Measurement time points**: baseline and immediately after the intervention  **Parental feeding practices**: parental feeding practices (fat and weight-based restriction, overt control, instrumental feeding, encouragement, monitoring, pressure to eat, emotional feeding and covert control)  **Other**: knowledge of intervention’s key points | **Intervention objectives:** promote parental responsive feeding practices  **IG** (*Happier Meals*): 20-minute vicarious learning video highlighting three mealtime problems: preschoolers not eating enough, resisting new foods and displaying picky eating. Key points of the video focused on: creating a healthy food environment, trusting children to make their own food choices, asking children help in preparing meals, modeling healthy eating and offering new foods 10-15 times.  **CG**: 20-minute TED talk about the education system |
| Sun et al. (2017)  EUA  *5-4-3-2-1-0 Program* | To examine the feasibility and efficacy of the intervention through a RCT (vs. a minimal intervention condition) to improve the health behaviors of low-income, overweight or obese Chinese mothers and their children. | Mothers of preschool children (3 to 5 years); Chinese mothers with BMI ≥ 23 or waist circumference > 31.5 inches.  **IG**: N=16 (analyzed: N=16)  **CG**: N=16 (analyzed: N=16) | **Measurement time points**: baseline, 3 and 6 months after the baseline  **Parental feeding practices**: child-feeding behaviors (pressure to eat, restrictions and monitoring of child’s eating); family eating and activity habits (stimulus exposure, eating related to hunger, eating styles)  **Other**: mother’s weight, height, BMI and waist circumferences; child’s weight and height; family health behaviors, objectively measured physical activity; feeding and perceived responsibility; parent and child’s weight status; weight concerns; child’s food preferences; family eating and activity habits (activity level); mother’s self-efficacy for promoting healthy eating, physical activity and limiting noncore foods | **Intervention objectives:** improve the health behaviors of mothers and their children (improve health knowledge and motivation will increase skills needed to affect behavioral change and to improved healthy behavior and outcomes: nutrition, screen time, physical activity)  **IG** (*5-4-3-2-1-0 Program)* : Eight weekly, 30-minute modules covering several topics: introduction, energy balance to maintain a healthy weight, feeding the family, grocery shopping, physical activity, screen time, smart parenting, and maintaining healthy weight for life. Modules were adapted from three existing programs (“*We Can!*”, “*5-4-3-2-1-Go!*” and “*5-2-1-0 Let’s Go!*”).  **CG**: Eight weekly mailings of printed health information about food safety, choking hazards, oral health, immunizations, appropriate antibiotic use, injury prevention and disaster preparation. |

Legend: CG = control group and IG = intervention group
