# Supplementary material for: Changing parental feeding practices through web-based interventions: A systematic review and meta-analysis
Source: PLoS One. 2021 Apr 28;16(4):e0250231. doi: 10.1371/journal.pone.0250231 (PMC8081248; doi:10.1371/journal.pone.0250231)
Supplement: Attachment — Submitted filename: Response to reviewers.docx [file pone.0250231.s007.docx]

Response to reviewers, 26.03.2021

**Dear editor:**

**Thank you very much for the detailed and careful review. We made all the changes requested by the reviewers. The changes in the text are marked in blue, and below we answer all comments, indicating the changes made and providing additional explanations about the highlighted issues.**

Journal Requirements:

Please review your reference list to ensure that it is complete and correct. If you have cited papers that have been retracted, please include the rationale for doing so in the manuscript text, or remove these references and replace them with relevant current references. Any changes to the reference list should be mentioned in the rebuttal letter that accompanies your revised manuscript. If you need to cite a retracted article, indicate the article’s retracted status in the References list and also include a citation and full reference for the retraction notice.

**Authors’ response: The reference list was reviewed. Although no major changes were performed (e.g., add or exclude references) since the last revision, in some cases, the article number/pages were missing. We have highlighted in blue all the changes made.**

Reviewer #1: Thank you for addressing the feedback provided, and I note your efforts to minimize the length and clarity of the article. A few further grammatical issues were noted:

| Page 24, Line 405 – There is some confusion regarding objectives versus research questions – either frame these as questions (as per the introduction), or change your terminology here to ‘objectives’. | We agree with your suggestion and changed these sentences to formulate them into questions |
| --- | --- |
| Page 25, Line 423 – Some feeding practices have been italicised while others have not – suggest italicising all feeding practices consistently for ease of reading | Thank you for noticing this issue. We revised and italicized all feeding practices in the text. We also noted that *Food availability/accessibility* practice was not always written in the same way, so we corrected the expression in the text to be according to the O’Connor categorization. |
| Page 27, Line 470 – ‘However, the Goal Setting was the BCT…’ – remove the first ‘the’ | The sentence was corrected. |
| Page 28, Line 480 – Do you mean to say here that there should be a balance between collecting enough information to allow for tailoring and not burdening parents with excessive data collection / questions? The last part of the sentence is a little unclear and perhaps could be reworded (i.e. detailed records sounds like you are presenting them with records, whereas I think you mean collecting records from them?) | Thank you for your suggestion. We agree that the sentence can be improved, so we changed to: Researchers must decide on the right balance between collecting enough information to allow for tailoring and not burdening parents with excessive data collection and records. |
| Page 29, Line 503 – Rather than ‘can be relevant’, perhaps ‘is suggested’ | We agree with your suggestion and changed the sentence. |

Reviewer #2: I would like to compliment the authors for the revisions they made. I have no further remarks and I think this piece will importantly contribute to the literature.
